# Collagen Cross- Linking for Paediatric Keratoconus

**DOI:** 10.2174/1874364101711010211

**Published:** 2017-07-31

**Authors:** Georgios D. Panos, Nikolaos Kozeis, Miltiadis Balidis, Marilita M. Moschos, Farhad Hafezi

**Affiliations:** 1 Department of Ophthalmology, Ipswich Hospital NHS Trust, University of Cambridge, Ipswich, Suffolk, UK; 2 Ophthalmica Institute, Thessaloniki, Central Macedonia, Greece; 3 Department of Ophthalmology, School of Medicine, National and Kapodistrian University of Athens, Greece; 4 ELZA Institute, Zurich, Switzerland; Faculty of Medicine, University of Geneva, Geneva, Switzerland; Faculty of Medicine, University of South California, California, USA

**Keywords:** Paediatric keratοcοnus, Cοllagen crοss- linking, Biometry, Cοrnea

## Abstract

**Background:**

Since the late 1990s corneal crosslinking (CXL) has been proposed as a new treatment option which can stop progression of keratoconus with promising results in adults.

**Objective:**

Keratoconus presents a higher rate and faster progression in paediatric patients and for this reason prompt and effective treatment is essential. Due to its success in adult keratoconus patients, CXL has been recently applied to children in order to stop or slow progression of keratoconus in paediatric patients.

**Conclusions:**

This article will present an update of the literature on the topic of CXL in this age group.

## 1 INTRODUCTION

Keratoconus is a progressive, bilateral, and asymmetric non-inflammatory corneal ectasia [1]. The disease traditionally manifests in the 2^nd^ decade of life when the cornea presents an increasingly conical shape, secondary to its biomechanical instability, which leads to irregular astigmatism and subsequent reduced visual acuity [1-3].

Traditional management of keratoconus consists of visual rehabilitation by means of spectacles, contact lenses and intracorneal ring implants for early to moderate stages and lamellar or penetrating keratoplasty in advanced stages [1, 4-6].

The introduction of corneal collagen cross-linking (CXL) by Wollensak *et al.* has changed the management of keratoconus [7]. CXL is a technique that uses the photochemical reaction between the Ultraviolet A (UVA) light and riboflavin within the corneal stroma and leads to the development of chemical bonds between collagen fibrils strengthening the cornea and slows or stops the progression of keratoconus and other corneal ectasia [8, 9]. Following its success in adult patients, CXL has been recently used for the treatment of paediatric patients with keratoconus [10-14].

## 2 CXL PROCEDURE

Standard CXL as described by Wollensak *et al.* is performed after removal of the central 7-9 mm of the epithelium using isotonic riboflavin 0.1% 20% and dextran solution every 5 minutes for 30 minutes. Ultraviolet-A irradiation (370 nm, 3 mW/cm^2^) is performed during 30 minutes and isotonic riboflavin solution is re-applied every 5 minutes. Finally, a bandage lens is placed and oral pain medication and antibiotic eye drops are prescribed [7]. Since, this first report there is a large number of publications in the literature reporting safety and efficacy of CXL in the treatment of keratoconus

and other corneal ectatic conditions (ex. pellucid marginal degeneration) [15-19].

These studies have provided evidence that CXL is effective in slowing or stopping keratoconus progression and may even improve patients vision by inducing corneal flattening and reduction in irregular astigmatism. Moreover, CXL (epithelium-off Dresden protocol) was found to be safe for corneal endothelium and intra-ocular structures when inclusion criteria are fulfilled (corneal thickness at least 400 um) with an acceptable rate of complications [15, 16].

Apart from the standard epithelium-off Dresden protocol, some clinicians have elected to perform CXL with the epithelium intact or partially disrupted or with the use of femtosecond-created intrastromal pockets, in an attempt to reduce post-operative discomfort and accelerate visual recovery [20-22]. The use of repeated applications of tetracaine 1% to try to loosen epithelial tight junctions has also been described [20]. Clinical studies have shown encouraging results however it remains controversial whether these novel protocols are as efficient as the standard epithelium-off one [23-27].

Standard treatment protocol utilizes UVA energies of 3 mW/cm^2^ and requires 30 min of UVA exposure to achieve the desired clinical effect as it is described above. It has been theorized that by increasing the UVA fluence while simultaneously reducing the exposure time, the same sub-threshold cytotoxic corneal endothelial UVA dosage can be administered, thereby maintaining efficacy and safety, but with a reduced treatment time. Cinar *et al.* in a study of 23 eyes showed that accelerated CXL produced a significant reduction in topographic keratometry values and an improvement in corrected distance acuity [28]. Kanellopoulos in a randomized, prospective study using a UVA power of 7 mW/cm^2^ for 15 min compared to 3 mW/cm^2^ for 30 min has demonstrated similar clinical results as the standard technique in terms of ectasia stabilization without any adverse effects associated with the higher fluence, shorter duration treatments [29].

## 3 CXL IN PAEDIATRIC PATIENTS

Keratoconus is most frequently diagnosed in young adults, however corneal changes (ex. ectasia) start much earlier [1]. It is well documented that keratoconus in paediatric patients presents a higher rate and speed of keratoconus progression compared to adults and is more aggressive [13, 30-35]. This may lead to a faster visual deterioration in this group of patients and affect the social and educational development and consequently their quality of life. Treating keratoconus at an earlier age is more beneficial than waiting until patients have more advanced disease requiring corneal transplantation. As the prognosis of corneal transplantation in children is poorer than in adults [36], a treatment to halt the progression, before corneal graft is necessary, could be of great benefit. CXL is effective and safe in halting the progression of keratoconus in adults. For this reason, CXL has been recently applied and evaluated in children.

Arora *et al*. [10], in their prospective study applied standard CXL in 15 eyes of 15 pediatric keratoconus patients (10 to 15 years) with moderate keratoconus in 1 eye and advanced disease in the fellow eye. At the end of the follow-up period (1 year), mean uncorrected distance visual acuity (UDVA) improved significantly from 1.00 ± 0.30 (20/200) to 0.72 ± 0.29 (20/100) logMAR (P=.035) and mean corrected distance visual acuity (CDVA) from 0.56 ± 0.21 (20/70) to 0.30 ± 0.15 (20/40) logMAR (P=.003). Mean change in apical K (1.01 ± 2.40 diopters) was also significant (P=.004). No significant complications were noted.

Vinciguerra *et al*. [12], in a prospective, interventional study included 40 eyes of 40 paediatric patients which underwent CXL. Mean logMAR baseline UDVA and CDVA were 0.79 ± 0.21 and 0.39 ± 0.10, respectively. Mean UDVA and CDVA at 2 years were 0.58 ± 0.18 and 0.20 ± 0.09, respectively. The improvement for both UDVA and CDVA was significant throughout the postoperative follow-up (P < .05). Mean baseline simulated keratometry was 46.32 D in the flattest meridian and 51.48 D in the steepest meridian; at 2 years, the values were 45.30 D (P = .04) and 50.21 D (P = .07), respectively. Moreover, for a 3-mm pupil, there was a significant reduction (P < .05) in whole eye (total), corneal, higher-order, and astigmatic wavefront aberrations at 24 months. A significant difference (P < .05) in total coma and total spherical aberration 2 years after CXL also was observed.

Caporrossi *et al*. [11], conducted a prospective nonrandomized phase II open trial (the “Siena CXL Pediatrics”) involving 152 patients aged 18 years or younger (10-18 years) with a follow-up of 36 months. UDVA and CDVA increased by +0.18 and +0.16 Snellen lines respectively in the thicker group (corneal thickness >450 μm) and +0.14 and +0.15 Snellen lines, respectively, in the thinner group (corneal thickness <450 μm). Topographic results showed statistically significant improvement in K readings and asymmetry index values. Coma reduction was also significant.

Our research group conducted a retrospective study 59 eyes from 42 children and adolescents (aged 9 to 19 years) with confirmed keratoconus with up to 3 years follow up [13]. Fifty-two of the 59 eyes enrolled in this study showed progression, corresponding to a progression rate of 88%. Forty-six eyes were treated by CXL. Maximal keratometry, CDVA, and KI showed significant changes over the follow-up period. However, significant Kmax reduction observed up to 24 months after CXL lost significance at 36 months. They proposed that awaiting documentation of progression is not mandatory and CXL in children and adolescents should be performed as soon as the diagnosis has been made. Zotta *et al.* in their retrospective case series evaluated the outcomes of CXL in paediatric patients with bilateral progressive keratoconus [37]. Four paediatric patients (eight eyes) with progressive keratoconus aged 14.0±2.2 years (range: 11 to 16 years) were included with a follow-up period of 36 months. All eyes underwent CXL in accordance with the standard Dresden protocol. Stabilization of K1 and K2 was demonstrated in all cases throughout follow-up while visual acuity improved in six eyes and remained stable in the remaining two eyes.

Magli *et al*. [23] conducted a comparative analysis of standard CXL (epi-off) and trans-epithelial CXL (TE CXL) protocols in a retrospective comparative evaluation of 37 eyes of 29 patients (12-18 years). In the epi-off CXL group (19 patients, 23 eyes; mean age, 14.75 ± 2.1 years), a significant improvement at month 12 was present for Kmax [-1.11 diopters (D), P = 0.01], Kmin (-3.2 D, P = 0.001), mean K (-1.47 D, P = 0.01), surface asymmetry index (-0.64 D, P = 0.001), inferior-superior symmetry index (-0.54 D, P = 0.01), index of height asymmetry (-2.97, P = 0.03), and anterior elevation at the thinnest location (-2.82 D, P = 0.01) and at the apex (-2.27 D, P = 0.01). Postoperative corneal oedema lasted 3 months in 16 eyes (69.5%) and more than 6 months in 2 eyes (8.7%). In the TE-CXL group (10 patients, 14 eyes; mean age, 15 ± 4.2 years), a significant improvement at month 12 was present for Kmax (-1.14 D, P = 0.02), Kmin (-2.04 D, P = 0.01), mean K (-1.63 D, P = 0.01), surface asymmetry index (-0.86 D, P = 0.001), inferior-superior symmetry index (-0.55 D, P = 0.001), index of height asymmetry (-2.95, P = 0.01), and anterior elevation at the thinnest location (-2.96 D, P = 0.01) and at the apex (-2.19 D, P = 0.01). No postoperative corneal oedema after TE-CXL was observed. Changes at month 12 from baseline were not significantly different between the 2 groups (P > 0.05). TE-CXE was significantly less painful than epithelium-off CXL.

On the other hand, Buzzonetti and Petrocelli [27], performed a prospective analysis of TE CXL for paediatric keratoconus (8 to 18 years age) in 13 eyes of 13 patients and demonstrated that despite CDVA improvement, transepithelial CXL does not effectively halt keratoconus progression in children compared to standard CXL.

Salman conducted a prospective, comparative study including 22 eyes of 22 patients younger than 18 years with bilateral keratoconus [38]. They had transepithelial CXL with the use of transepithelial riboflavin. The other eye was used as a control and was treated conservatively. After transepithelial CXL, the improvement in the mean UDVA was statistically significant (from 0.95 ± 0.34 logMAR to 0.68 ± 0.45 logMAR) (P<.05). No eye lost lines of preoperative UDVA; 1 eye lost 1 line of preoperative CDVA. The mean simulated keratometry (K) decreased by a mean of 2.03 diopters (D), with mean flattening of the apical K by 2.20 D; both results were statistically significant (P<.05). In the control group, the simulated K increased by a mean of 0.59 D (P>.05), with mean steepening of the apical K by 2.9 D (P<.05) suggesting that preliminary results of transepithelial CXL in children with keratoconus were encouraging, with no evidence of progression of keratoconus over 12 months.

Recently McAnena and 'Keefe published a retrospective study in order to report the visual, refractive, and tomographic outcomes of corneal collagen crosslinking (CXL) in paediatric patients with keratoconus [39]. They demonstrated that CXL effectively stabilized uncorrected visual acuity, refractive indices, and keratometry values at 1 year, while improving best-corrected visual acuity.

A summary of the studies is depicted in (Table **1**).

## CONCLUSION

Keratoconus presents a higher rate and faster progression in paediatric patients and for this reason prompt and effective treatment is essential. Despite limited evidence in paediatric patients, CXL may be considered in the management of progressive paediatric keratoconus when comparing the risks of visually significant complications from CXL and the risk of visual loss from accelerated progression of keratoconus in young patients, it is clear that CXL should be offered without waiting for progression. Standard epithelium-off CXL protocol should be applied, as there is no evidence yet that TE – CXL can provide similar efficiency and safety. Parents should be aware of possible adverse effects, short lasting effect and need for re-treatment in cases with aggressive form. Future research should focus on possible different protocols regarding the power and the duration of the technique, and on the timing of the treatment in order to improve long term outcomes and reduce complications. These results are anticipated with great interest.

## Figures and Tables

**Table 1:** Summary of published studies regarding outcomes of corneal collagen cross linking in paediatric keratoconus.

**Study **	**Subjects**	**Age **	**Design**	**Follow-up**	**CXL Protocol **	**Outcome **
Arora *et al*.	15	10-15	Prospective	12 months	Standard	Improvement
Vinciguerra *et al*.	40	9-18	Prospective	24 months	Standard	Improvement
Caporrossi *et al*.	152	10-18	Prospective	36 months	Standard	Improvement
Chatzis *et al*.	42	9-19	Retrospective	36 months	Standard	Initial improvement, late progression
Zotta *et al*.	4	11-16	Retrospective	36 months	Standard	Improvement or stabilization
Magli *et al*.	29	12-18	Retrospective	12 months	Epi-on and Epi-off	Improvement for both groups
Buzzonetti and Petrocelli	13	8-18	Prospective	18 months	Epi-on	Progression
Salman	22	13-18	Prospective	12.05 months (mean)	Epi-on	Improvement
McAnena and O'Keefe	14	13-18	Petrospective	12 months	Standard	Stabilization
